# Mir-34a Mimics Are Potential Therapeutic Agents for p53-Mutated and Chemo-Resistant Brain Tumour Cells

**DOI:** 10.1371/journal.pone.0108514

**Published:** 2014-09-24

**Authors:** Yuen Ngan Fan, Daniel Meley, Barry Pizer, Violaine Sée

**Affiliations:** 1 University of Liverpool, Department of Biochemistry, Centre for Cell Imaging, Liverpool, United Kingdom; 2 UPRES EA 4245 « Cellules Dendritiques, Immunomodulation et Greffes », Université François-Rabelais, Tours, France; 3 Alder Hey Children’s NHS Foundation Trust, Liverpool, United Kingdom; University Hospital of Navarra, Spain

## Abstract

Chemotherapeutic drug resistance and relapse remains a major challenge for paediatric (medulloblastoma) and adult (glioblastoma) brain tumour treatment. Medulloblastoma tumours and cell lines with mutations in the p53 signalling pathway have been shown to be specifically insensitive to DNA damaging agents. The aim of this study was to investigate the potential of triggering cell death in p53 mutated medulloblastoma cells by a direct activation of pro-death signalling downstream of p53 activation. Since non-coding microRNAs (miRNAs) have the ability to fine tune the expression of a variety of target genes, orchestrating multiple downstream effects, we hypothesised that triggering the expression of a p53 target miRNA could induce cell death in chemo-resistant cells. Treatment with etoposide, increased miR-34a levels in a p53-dependent fashion and the level of miR-34a transcription was correlated with the cell sensitivity to etoposide. miR-34a activity was validated by measuring the expression levels of one of its well described target: the NADH dependent sirtuin1 (SIRT1). Whilst drugs directly targeting SIRT1, were potent to trigger cell death at high concentrations only, introduction of synthetic miR-34a mimics was able to induce cell death in p53 mutated medulloblastoma and glioblastoma cell lines. Our results show that the need of a functional p53 signaling pathway can be bypassed by direct activation of miR-34a in brain tumour cells.

## Introduction

Medulloblastoma (MB) is a common malignant paediatric brain tumour, developing in the posterior fossa of the brain and comprising 15–20% of paediatric tumours of the central nervous system (CNS) [1]. MB arises from neural stem cells or granule-cell progenitors of the cerebellum and in around 30% of cases metastasises to other areas of the CNS via the cerebrospinal fluid. MB has recently been sub-classified based on the differences in their transcriptome, with the four main subgroups being: WNT, SHH, Group 3 and Group 4 [2]. The current treatment for MB includes surgery, cranioradiotherapy and chemotherapy. However, treatment is frequently associated with significant neuro-psychological and physical disabilities [1], [3] and chemotherapy remains the only treatment option available for younger patients following surgery. A related problem is chemoresistance, which has previously been reported in patients and MB cell lines [4]–[7]. It has been shown to be associated with altered drug metabolism [4], [6] or genetic mutations affecting essential signalling pathways, such as NF-kappaB and/or p53 [7], [8].

The p53 pathway plays a vital role in maintaining genomic integrity by transactivating target genes involved in cell cycle arrest, DNA repair, apoptosis and senescence [9], [10]. For this reason, p53 activating compounds such as DNA damaging agents are attractive candidates for chemotherapy. The chemotherapeutic cocktail combination, used for treating MB in the clinic [11], [12], fully relies on a functional p53 activation for their cytotoxic effect. For example, etoposide, a topoisomerase II inhibitor, triggers accumulation of double stranded breaks within DNA and subsequent activation of p53 and cell death. Whilst p53 mutations are enriched across all MB subgroups, p53 mutations in the SHH group correlate with poor survival and treatment failures [13]. Hence, novel therapeutic agents, capable of triggering cell death by activating pro-apoptotic signalling downstream of p53, are crucially needed to kill p53 mutated medulloblastoma cells.

Active p53 will transcribe a wide range of coding mRNA as well as noncoding microRNAs (miRNAs). miRNAs are negative regulators of gene expression, controlling genes involved in many biological processes, ranging from larval development, cell differentiation, proliferation and apoptosis [14]–[18]. They down-regulate gene expression by perfect or partial complementary binding to the 3′-untranslated region (3′-UTR) of target mRNA, promoting its degradation or preventing protein translation [19], [20]. Among many identified miRNAs, miR-34a is associated with a variety of cancer types [21] and is a well described transcriptional target for p53 [22]. miR-34a targets include factors required for cell cycle progression, anti-apoptotic proteins and proteins involved in invasion [23]–[25]. Hence, miR-34a functions as a tumour suppressor, therefore its activation could potentially achieve tumour regression without the need of a functional p53 pathway.

Here, we investigated the expression of miR-34a in MB cells in response to drug treatment and the correlation between miR-34a induction and MB cell response to chemotherapeutic treatment. We demonstrated that miR-34a upregulation upon etoposide exposure is associated with increased cell sensitivity to etoposide in MB cell lines. Inhibition of sirtuin-1 (SIRT1), a well described target of miR-34a [26] was not enough to trigger cell death. However, miR-34a mimic expression could directly induce cell death in p53 mutated and hence chemo-resistant MB cells, thus bypassing upstream p53 activation. This beneficial role of miR-34a mimic in activating cell death was also confirmed in the adult brain tumour glioblastoma cell lines (GBM) mutated in p53.

## Materials and Methods

### Reagents

Etoposide (cat# E1383), Nicotinamide (cat# 479865-U) and Sirtinol (cat# s7942) were from Sigma-Aldrich Company Ltd (Dorset, UK). Cisplatin (cat# 440-040) and metothrexate (cat# 440-045) were from Enzo Life Sciences UK Ltd (Exeter, UK). EX527 (cat# 2780) was from Tocris bioscience (R&D systems, UK). Tissue cell culture media were supplied by Gibco Life Technologies and foetal calf serum by Harlan Seralab (UK). Cyclophilin A (cat# Ab3563), actin (cat# Ab8226), SIRT1 antibody (cat# Ab13749), and HRP-anti-mouse (cat# Ab6808) antibodies were from Abcam (UK). p53 BC-12 antibody (cat# SC126) was from Santa-Cruz Biotechnology (Texas, USA). c-Myc antibody (cat# 9402), Bcl-2 (cat# 2870) and HRP-anti-rabbit (cat# 7074) antibody were from Cell Signalling Technology (MA, USA). miR-34a mimic (cat# C-300551-07), control (cat# CP001000-02-05) were purchased from Dharmacon (now GE Healthcare, UK). siRNAs targeted for p53 (cat# 1299001) and scrambled siRNA were from Invitrogen.

### Cell culture

D283-MED (medulloblastoma) [27], U87MG and T98G (glioblastoma) were purchased from ATCC. Medulloblastoma MHH-Med1 cells and MEB-Med8A [28] cells were kindly provided by Prof T. Pietsch (University of Bonn, Germany). D283-MED and MHH-Med1cells were maintained in modified Eagle’s medium (MEM) with 10% FCS, 1% non-essential amino acid and 1% sodium pyruvate. MEB-Med8A cells were maintained in Dulbecco’s MEM (DMEM) with 10% FCS. U87MG and T98G cells were maintained MEM with 10% FCS and 1% sodium pryruvate. Cells were cultured at 37°C and 5% CO_2_.

### Cell transfection

For plasmid expression, cells were transfected with pMT-p53-dsRedXP (originally given by Dr G Lahav, Harvard University, Cyan Fluorescent protein was replaced by dsRedXP) and pMDM-2-MDM-2-YFP (from Dr G Lahav, Harvard University) using Fugene HD (Roche, UK) at 4∶2 reagent per µg DNA ratio for 24 hours. For siRNA transfection, cells were transfected with siRNA directed for p53 [100 nM] or with non-specific siRNA as a negative control [nM] using HiPerfect (Qiagen) for 48 hours expression. For miR-34a mimic expression, cells were transfected with miR-34a mimics [100 nM] or with a house keeping (GAPDH) transfection control using HiPerfect (Qiagen) and expressed for 72 hours before MTS assay or 48 h before harvesting for western-blot.

### Quantitative Real time PCR (qPCR)

RNA were purified with miRNeasy (Qiagen). 1 µg of RNA were used for cDNA synthesis using 1st strand synthesis kit and according to the manufacturer’s protocol (Invitrogen). Quantitative qPCR was performed using a LightCycler 480 instrument (Roche) containing 10 µl of SYBR^−^Green mix, forward and reverse primers [250 nM each] and 200 ng of cDNA in each reaction. All reactions were performed in triplicates and qPCR program was as follow: 50°C 2 min, 95°C 10 min, (95°C 5 sec, 60°C 30 sec)×45, automated dissociation steps. Primers sequences were: Cyclophilin A: Forward: GCTTTGGGTCCAGGAATGG; Reverse: GTTGTCCACAGTCAGCAATGGT; MDM2: Forward: GCAAATGTGCAATACCAACA; Reverse: CTTTGGTCTAACCAGGGTCTC; SIRT1: Forward: TTTGGAAATGTTTCAGTTGCTTTA; Reverse: CACTCTCCCCAGTAGAAGTACCAT; miR-34a: Forward: TGGCAGTGTCTTAGCTGGTTGT; Reverse: Universal Primer (Invitrogen).

### Cell viability MTS assays

D283-MED, MEB-Med8A, U87MG and T98G cells were seeded on a 96 well culture plate 24 hours prior drug treatments. The cells were treated with etoposide [20 µM], cisplatin [5 µM], methotrexate [5 µM], nicotinamide [10–100 mM], EX527 [50 or 100 µM] and sirtinol [50 or 100 µM] or left untreated (control) at indicated time points in replica of 6. CellTiter 96 Aqueous One Solution (Promega) was added to the plate and incubated for 1 to 2 hours at 37°C at the end of each treatment time point. Measurement was obtained with a plate reader at 492 nm (Multiskan, Thermo Scientific).

### Confocal microscopy

Transfected cells on glass bottom dishes (Iwaki, Asahi Techno Glass) coated with poly-ornithine were incubated on the microscope stage at 37°C, 5% CO_2_ of a LSM 510 (Zeiss) with a 63x Plan Apochromatic oil immersion objective (NA 1.4). YFP- tagged protein was excited using an Argon ion laser (488 nm) and dsRedXP-tagged protein was excited using a green-neon laser (543 nm). Emitted light was detected through a 505–550 nm band-pass filter (YFP) and a 560 nm long pass filter through a dichroic mirror (Red fluorescence). Data was analysed using CellTracker v.6 software (http://dbkgroup.org/celltracker).

### Immunoblotting

D283-MED and MEB-Med8A cells were treated with etoposide as indicated. Protein extract and immunoblotting were performed as previously described [8] using the antibodies listed in the ‘Reagents’ section.

### Immunocytochemistry

Cells were seeded on glass bottom dishes (Iwaki, Asahi Techno Glass) and fixed with 4% paraformaldehyde for 15 minutes and blocked with 1% BSA, 0.1% Triton X-100 in PBS for 20 min. Cells were then incubated with SIRT1 antibody (1∶200; Abcam Ab13749) in blocking buffer for 1 hour. After 3 PBS washes, cells were incubated with anti-rabbit IgG FITC conjugate (1∶500; Invitrogen cat# A11008) for 30 minutes under light protection. After 3 PBS washes, cells were imaged using a LSM 710 confocal microscope (Zeiss). AQM Advance 6.0 software (Kinetic Imaging) was used for image analysis.

### Statistical analysis

Statistical significance test was performed using one-way ANOVA followed by Bonferroni test using built in statistical analysis in OriginPro 8.6.0 (OriginLab Corporation, USA). With the exception of qPCR data, which are not normally distributed, a non-parametric Krustal-Wallis ANOVA was used (OriginLab Corporation, USA).

## Results

### MiR-34a up regulation depends on a functional p53 and is correlated with cell sensitivity to chemotherapeutic drugs

**Figure 1:**
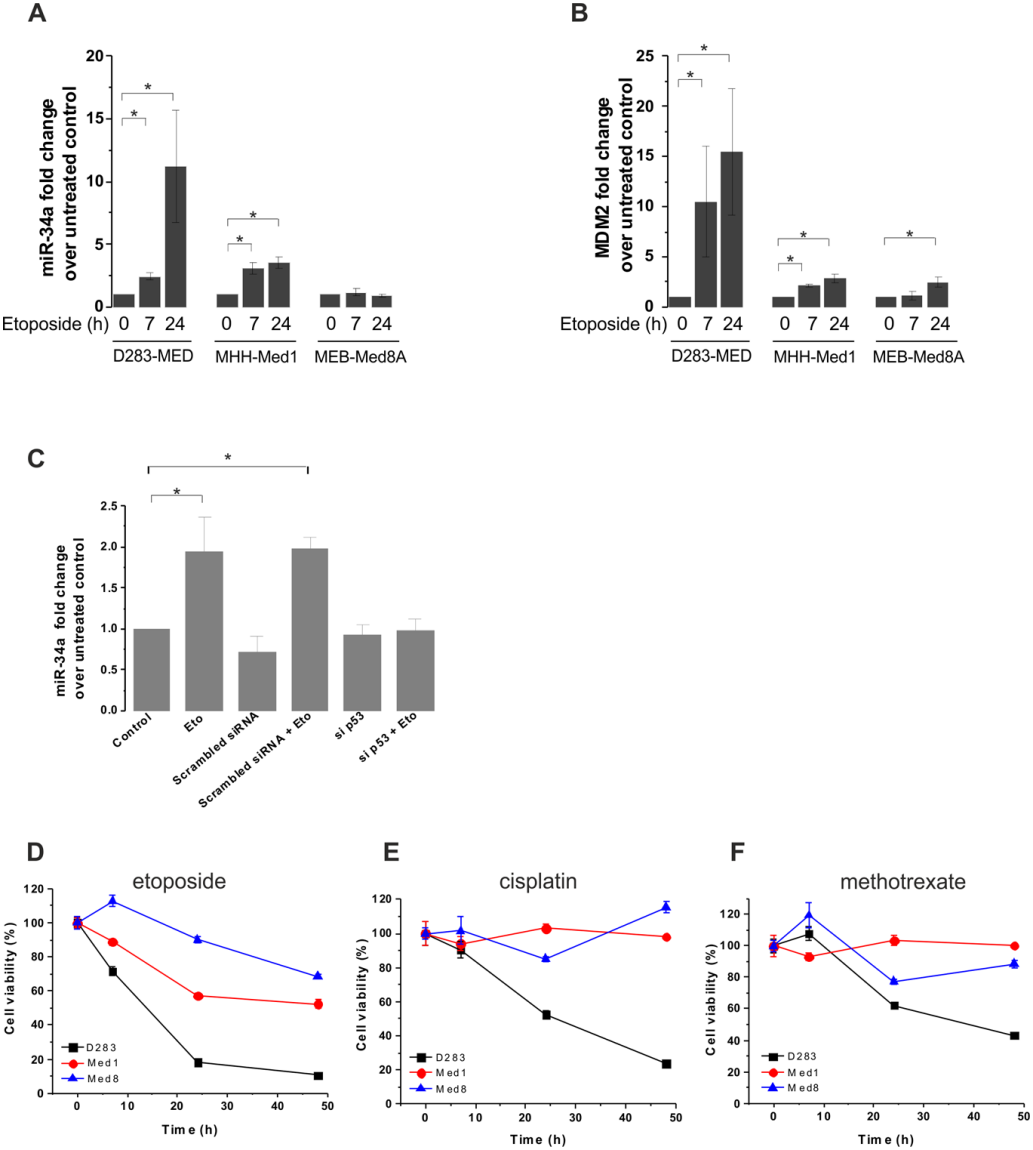
MB cell lines display different levels of MiR-34a induction, which correlate with sensitivity to chemotherapeutic drugs. (**A, B**) D283-MED, MHH-Med1 and MEB-Med8A cells were treated with [20 µM] etoposide for indicated time points and levels of miR-34a (A) and Mdm2 mRNA (**B**) were assessed by real time qPCR. Results were normalised to cyclophilin A and fold changes relative to the untreated control. See also Fig. S1A for a DMSO control. (**C**) D283-MED cells were transfected with siRNA directed to p53 or with non-specific siRNA as a negative control for 48 hours prior to etoposide treatment. The levels of miR-34a were assessed by qPCR as in (A). Data shown are the mean ± S.E.M of three independent experiments. (A–C) Kruskal-Wallis ANOVA test was performed (*indicates p<0.05). (**D**–**F**) Cell viability of D283-MED, MHH-Med1 and MEB-Med8A was measured by MTS assay upon treatment at indicated time points. (**D**) Etoposide [20 µM] (**E**) cisplatin [5 µM] (**F**) methrotrexate [5 µM]. The percentages of viable cells were relative to the untreated control. See also Fig. S1B for a vehicle DMSO control on cell death. Data shown are the mean ± S.E.M of three independent experiments.

MB cell lines have previously been shown to be resistant to chemotherapy intervention in the presence of mutations in the p53-dependent pathway [8], [13]. One strategy to overcome the effects of drug resistance caused by impaired p53 activation is to directly target downstream of p53, bypassing the need of its activation. p53 up-regulates a number of downstream targets, including pro-apoptotic genes to induce cell death. It is possible to specifically activate one of these targets; however, it is likely that triggering one individual downstream candidate will not be enough to induce a significant cell death response. For this reason, targeting a p53-dependent miRNA, which itself controls hundreds of mRNA/protein expression, could be more powerful. One such miRNA is miR-34a, which has been shown to be a downstream transcriptional target of p53 [22]. We first investigated if miR-34a was induced by etoposide in a range of MB cell lines previously used [29] and if this induction was correlated with induction of cell death. In D283-MED cells, miR-34a levels were increased upon etoposide treatment in a time dependent manner with up to 11 fold increase following a 24 hour exposure to etoposide (Fig. 1A). In comparison, miR-34a levels in MHH-Med1 cells were up-regulated by ∼3 fold only after 24 hours and no significant induction of miR-34a was observed for the MEB-Med8A cell line up to 24 hours of etoposide treatment (Fig. 1A). The kinetics and amount of miR-34a transcription were correlated with p53 transcriptional activity measured here by Mdm2 transcripts (Fig. 1B). DMSO was used at 1/1000 (v/v) for etoposide dilution; the absence of effects of DMSO on p53-dependent transcription are shown in Fig. S1A.

We further confirmed the essential role of p53 in etoposide-induced miR-34a. D283-MED cells were transfected with a siRNA directed to p53 or a non-specific siRNA as a negative control followed by etoposide treatment. We demonstrated that etoposide-induced miR-34a transcription was abolished in the presence of the p53-directed siRNA (Fig. 1C). Taken together, the lack of etoposide-induced miR-34 induction in the siRNA experiment and in MEB-Med8A cell line confirms that miR-34a transcription is dependent on p53 activity and that it cannot be activated in tumour cells bearing an impaired p53 pathway.

The lack of miR-34a transcription in MEB-Med8A cells was concurrent to the cell resistance to a range of chemotherapeutic agents (Fig. 1D–F). We assessed the cell viability of the 3 different MB cell lines treated with etoposide, cisplatin or methotrexate. A DMSO control for all cell lines is presented Fig. S1B. As previously observed by us and others [8], [30], the D283-MED was the most sensitive cell line, whereas MEB-Med8A was very resistant to etoposide (Fig. 1D). MEB-Med8A cell line showed a maximum of 30% of cell death after 48 hours of etoposide treatment compared to 90% in D283-MED cells. Moreover, D283-MED cell line was also the most sensitive to cisplatin and methrotrexate, whereas MEB-Med8A cells exhibited the strongest resistance to these drugs (Fig. 1D–F). MHH-Med1 cells had strong resistance to cisplatin and methotrexate with little cell death up to 48 hours treatment yet displayed an intermediate sensitivity to etoposide with ∼40% of cell death after 48 hours (Fig. 1D–F).

### MEB-Med8A cells have an impaired p53 signalling that cannot be restored by WT p53 expression

**Figure 2:**
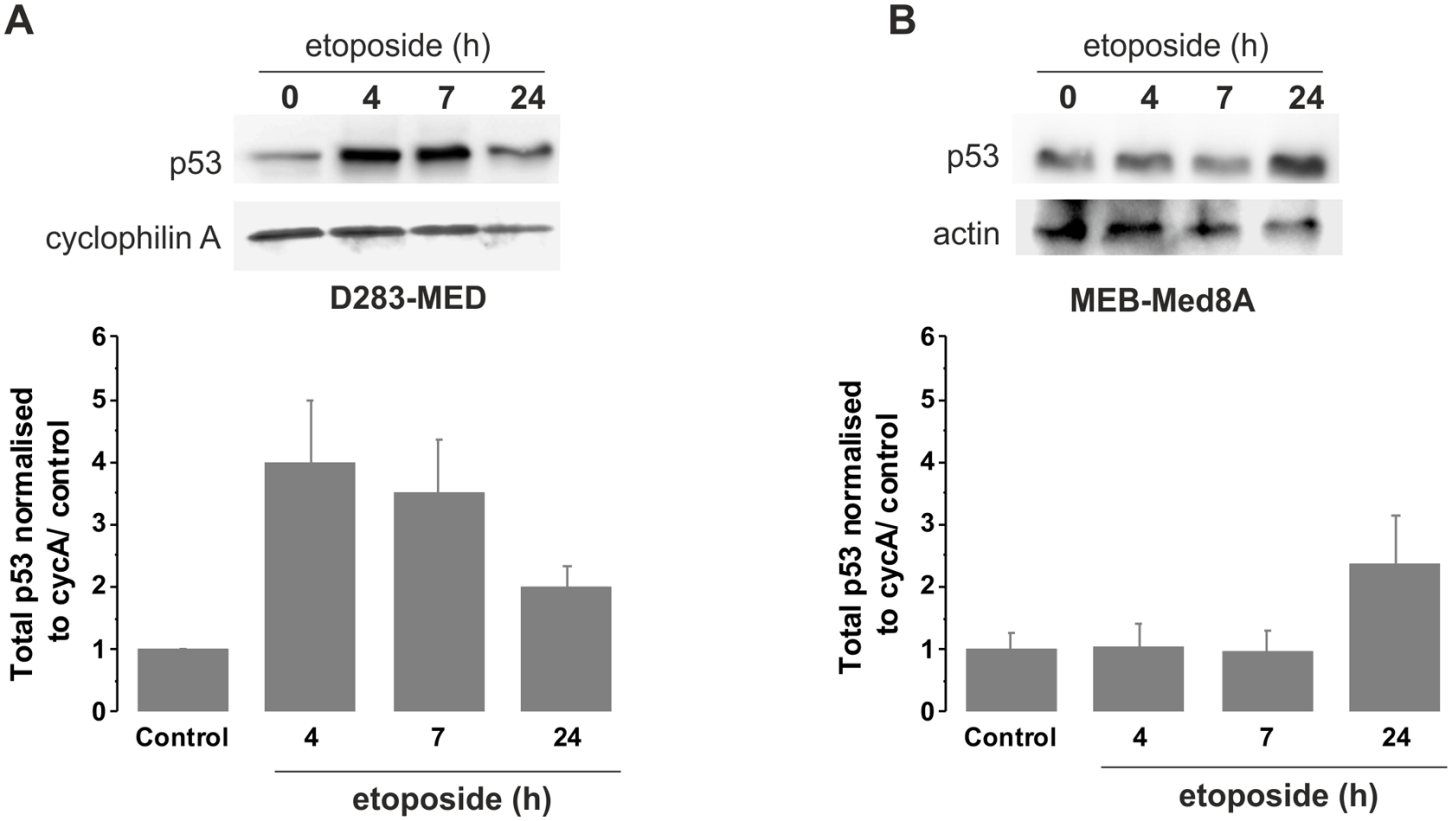
p53 activation is impaired in MEB-Med8A cells. (**A**) D283-MED cells were treated with [20 µM] etoposide for indicated times and the p53 protein levels were measured by western blot. (**B**) MEB-Med8A cells were treated with [20 µM] etoposide for indicated times and p53 protein levels were measured by western blot. 2 gels from independent experiments were quantified by densitometry analysis (AQM Advance 6 imaging software, Kinetic Imaging Ltd). The plot shown is the result of the quantification relative to cyclophilin A levels and normalised to t0 untreated control ± sd for each cell line.

**Figure 3:**
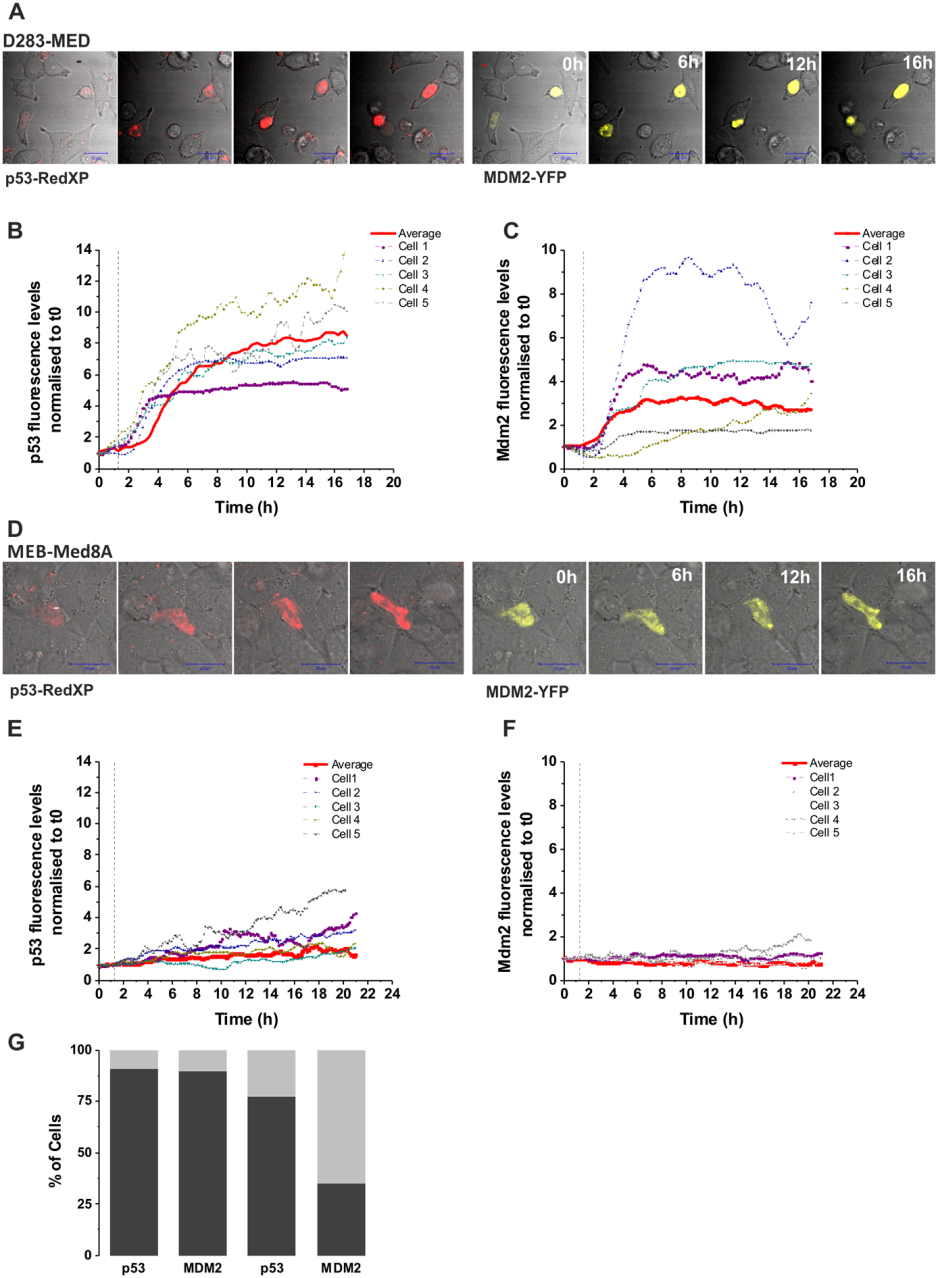
Expression of WT p53 does not restore p53 activity in MEB-Med8A cells. (**A–C**) D283-MED cells were co-transfected with p53-dsRedXP and MDM2-YFP and imaged using time lapse confocal microscopy. The time of etoposide stimulation is represented by the vertical dotted line. The level of p53 and MDM2 were assessed by measuring fluorescence intensity in single cells over time, which were normalised to the baseline fluorescence measured prior to etoposide addition. (B, C): Example of 5 single cell traces and the average fluorescent intensity (red line) of all cells are shown (N = 2, n = 33). (**D–F**) MEB-Med8A cells were co-transfected and imaged as in (A–C). (E–F): Example of 5 single cell traces and the average fluorescent intensity (red line) of all cells are shown (N = 2, n = 22). (**G**) A stack column showing the percentage of MB cells with p53 or MDM2 expression above threshold level upon etoposide treatment. Threshold was calculated as average intensity of untreated control +2 SD. D283 cells (N = 2, n = 33); Med8 cells (N = 2, n = 22).

We further investigated, in the most sensitive and resistant cell lines (D283-MED and MEB-Med8A), the induction of p53 upon a time course of etoposide. We observed that the p53 protein levels were weakly and slowly induced (2 fold increase at 24 h) in MEB-Med8A cells, yet a 4 fold increase was observed after 4 h etoposide in D283-MED cells (Fig. 2A, B), consistent with the p53 transcriptional activity measured in Fig. 1B. We hypothesised that the MEB-Med8A cells might be re-sensitised to etoposide by the expression of an exogenous full WT p53 protein. MB cells were transiently co-transfected with pMT-p53-dsRedXP and pMdm-2-YFP expressing plasmids. Time-lapse confocal microscopy showed a strong p53 induction above background levels upon etoposide stimulation in 91% of the D283-MED cells analysed (n = 33; Fig. 3A, B, G) and Mdm2 was also induced with a 30 min delay compared to p53 (90% of the cells; Fig. 3C, G). Examples of cell traces are shown on Fig. 3B–C. The delay of Mdm-2 induction is likely to be due to the time required for p53 to activate the Mdm-2-YFP promoter. Many cells underwent apoptosis or showed apoptotic morphology within 24 hours, due to their sensitivity to etoposide. In contrast, in MEB-Med8A cells, the p53 increase was considerably lower compared to D283-MED (Fig. 3D–F, G) and only 35% of the cells showing an increased p53 also had a Mdm-2 induction (Fig. 3F, G; n = 22). Also, p53 activation was much slower, with an average of 2.6 hours after stimulation for p53 increase above background and 4.8 hours for Mdm-2 to be compared with and 1.1 hours (for p53) and 1.6 hours (for Mdm-2) in the D283-MED cells (Fig. 3A–F). This indicated that not only p53 seems to be non-functional in the MEB-Med8A cell lines, but that it cannot be compensated by re-introduction of WT p53.

### miR-34a targets SIRT1 degradation upon etoposide treatment

**Figure 4:**
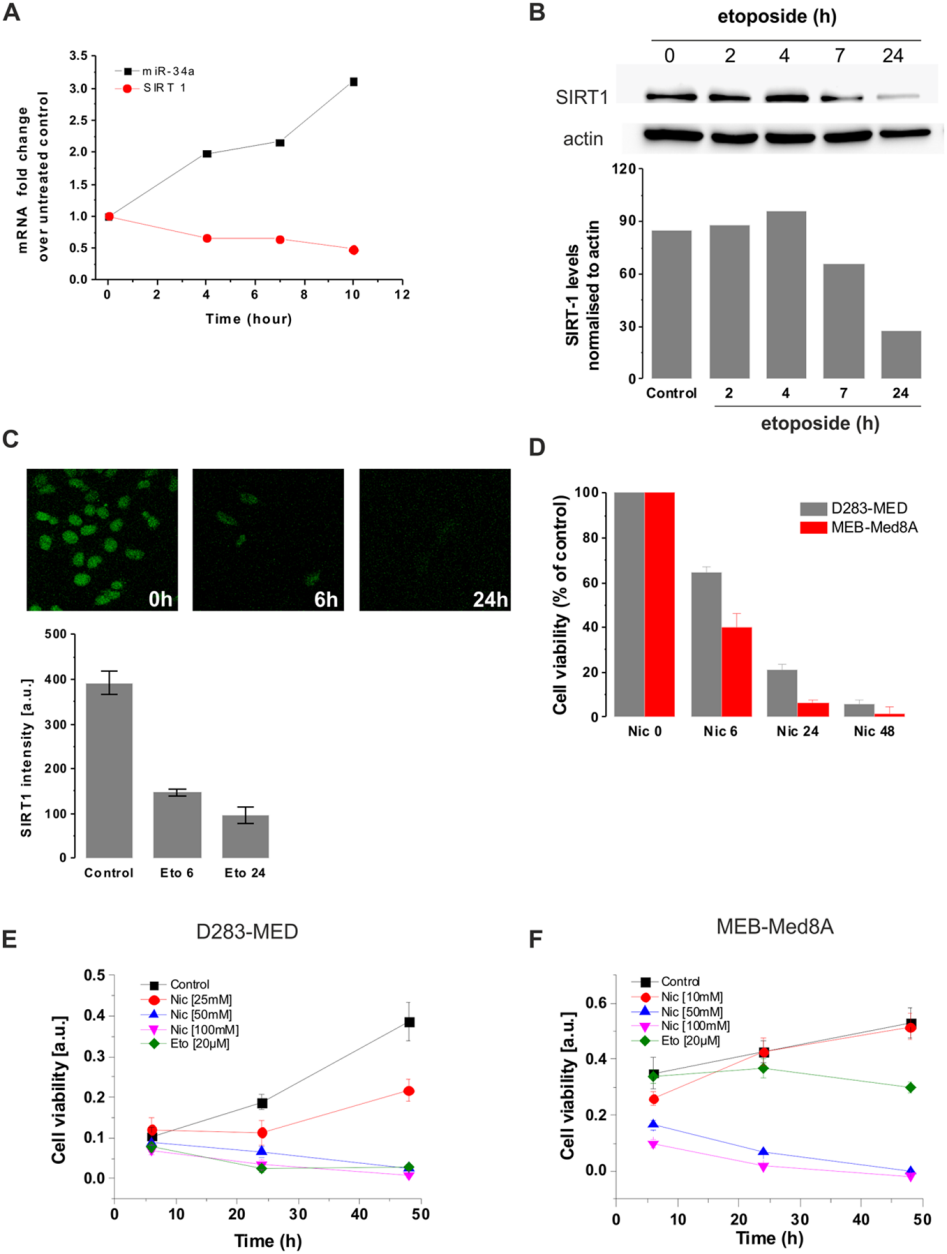
miR-34a expression correlates with down-regulation of SIRT1. (**A**) mRNA levels of SIRT1 and miR-34a were measured by qPCR upon [20 µM] etoposide treatment at indicated time points in D283-MED cells. (**B**) SIRT1 protein levels upon [20 µM] etoposide treatment were assessed by western blot. Band density was quantified by densitometry analysis. The blot shown is representative of four independent experiments. (**C**) SIRT1 levels and localisation were detected by immunofluorescence. Quantification was quantified by densitometry analysis (AQM Advance 6 imaging software) (**D**) D283-MED and MEB-Med8A cells were treated with [100 mM] nicotinamide for indicated time points. The percentage of cell viability was measured by MTS assay and normalised to the untreated control. Data shown are the mean ± S.E.M of three independent experiments. One-way ANOVA followed by Bonferroni test was performed (*indicates p<0.05). (**E, F**) Dose response of MB cells treated with nicotinamide. Cells were treated with nicotinamide at indicated concentrations and times and cell viability was measured by MTS assay. (**E**) D283-MED cells and (**F**) MEB-Med8A cells. Error bars shown are SD of 6 replicates.

The functional role of miR-34a induction was confirmed by looking at one of the miR-34a downstream target involved in cell survival: SIRT1 [26]. We measured SIRT1 mRNA and protein levels upon etoposide treatment to determine miR-34a activity. SIRT1 mRNA level decreased concomitantly to the increase of miR-34a upon etoposide treatment (Fig. 4A). We further examined SIRT1 protein levels by western blot and immunocytochemistry (ICC) and observed a decrease of protein levels upon etoposide exposure with no detectable levels after 24 hours of etoposide (Fig. 4B, C). Taken together, our data suggest that etoposide-induced miR-34a via p53 activation, correlates with the down-regulation of SIRT1 expression. We next explored SIRT1 pro-survival function in MB cells by using a SIRT1 inhibitor: nicotinamide. SIRT1 inhibition resulted in cell death for both p53 WT and p53 mutated cell lines (D283-MED and MEB-Med8A respectively; Fig. 4D) in a dose and time dependent manner (Fig. 4E, F). This suggests that it is possible to bypass p53 pathway to induce cell death. However, only high concentrations of nicotinamide could induce significant cell death and other SIRT1 inhibitors such as EX527 and sirtulin did not induce significant cytotoxic effect on D283-MED and MEB-Med8A cells (Fig. S2A–C). These results therefore question the specificity of SIRT1 inhibition and challenge the potential benefits for cell death induction of targeting a single p53 downstream protein.

### miR-34a mimics induces cell death in p53 mutated cells

**Figure 5:**
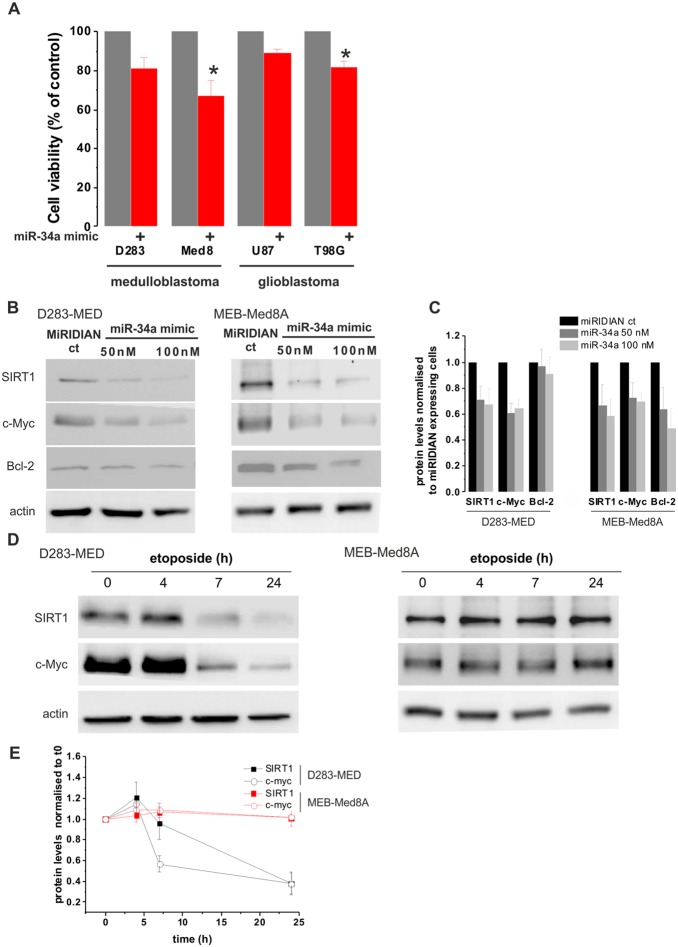
miR-34a-mimic induces cell death in p53 mutated MB and GBM cells. (**A**) D283-MED, MEB-Med8A MB, U87MG and T98G cells were transfected with [100 nM] miR-34a mimics or non-specific mimic miRIDIAN control and expressed for 72 hours prior to cell viability assessment by MTS assay. Cell viability was normalised to cells transfected with the non-specific mimic control. Data shown are the mean ± S.E.M of four independent experiments. One-way ANOVA followed by Bonferroni test was performed (*indicates p<0.05). (**B**) D283-MED and MEB-Med8A MB were transfected with [50 nM] or [100 nM] of miR-34a mimic or [50 nM] of non-specific miRIDIAN control for 48 h. Expression levels of SIRT1, c-Myc and Bcl-2 were assessed by western-blot. (**C**) 2 gels form independent experiments were quantified by densitometry analysis. The plot shown is the result of the quantification relative to actin levels and normalised to cells transfected with the non-specific miRIDIAN control ± sd. (**D**) D283-MED and MEB-Med8A MB were treated with [20 µM] etoposide for indicated time points. Expression levels of SIRT1 and c-Myc were assessed by western-blot. (**E**) 2 gels form independent experiments were quantified by densitometry analysis. The graph shown is the result of the quantification normalised to untreated cells at t0 ± sd.

Since miR-34a has the ability to regulate a large set of target genes, it might serve as a better p53 downstream effector than targeting SIRT1 to promote cell death. We hence tested the efficacy of using miR-34a mimic to induce cell death independent of p53 activation. MB cell lines were transfected with synthetic miR-34a mimic oligonucleotides for 72 hours. Interestingly, the miR-34a mimic induced ∼35% of cell death in the p53 mutated cell line MEB-Med8A previously insensitive to etoposide (Fig. 5A). The efficiency of miR-34a mimic to induce cell death in the p53WT D283-MED was lower with only 20% of cell death compared to control conditions. We further confirmed the potential role of miR-34a mimic in a wider context, by including adult brain tumour cell lines. We used, the glioblastoma (GBM): U87MG (p53 WT) and T98G (p53 mutated). We again achieved a higher cell death in the p53 mutated GBM cells compared to the p53 WT (20% to be compared to 10%; Fig. 5A). To further investigate the difference in the efficacy of miR-34a mimic to induce cell death in MEB-Med8A and D283-MED cells, the protein levels of three experimentally validated miR-34a oncogene targets: SIRT1 [26], c-Myc [31], and Bcl-2 [32] were measured. Figures 5B&C show that, in both D283-MED and MEB-Med8A cells, miR-34a mimic is able to trigger the down-regulation of SIRT1 and c-Myc, However Bcl-2 expression was only decreased in MEB-MED8A cells. The difference in the regulation of the anti-apoptotic Bcl-2 protein by miR-34a expression could explain the greater sensitivity of MEB-Med8A cells to cell death induced by miR-34a expression. The SIRT1 and c-Myc down-regulation by miR-34a mimic expression was also compared to etoposide treatment in both cell lines (Fig. 5D, E). As expected, etoposide had no effects on SIRT1 and c-Myc levels in MEB-Med8A cells, which is correlated with the absence of p53 activation and cell death induction by etoposide in these cells. In D283-MED cells, both proteins were downregulated upon etoposide treatment in line with the SIRT1 experiment shown in Fig. 4B. The decreased level of both proteins was much more pronounced for etoposide treated cells (about 60% loss after 24 h etoposide treatment) than upon miR-34a expression (about 30% loss). This result together with the cell viability data in Fig. 5A indicates that etoposide is more efficient to induce cell death than miR-34a expression in cells with a functional p53 signalling pathway. Nevertheless, our results clearly demonstrate that p53 activation can be bypassed and that the direct activation of the apoptotic pathway using miR-34a mimics can be achieved, especially in cell lines lacking p53 activity and previously shown to be resistant to chemotherapeutic agents.

## Discussion and Conclusions

The loss of *TP53* due to the loss of chromosome 17p and isochromosome 17q are the most prevalent chromosomal abnormalities observed in MB [33]–[35]. Mutations in *TP53* were found in 16% of MB and this is associated with a poor 5 year survival rate [36]. We have previously reported that p53 mutations in MB cell lines are also associated with increased drug resistance, which is likely to be, at least in part, responsible for the worse prognosis observed in patient with p53 mutations [8]. We here propose an alternative potential therapeutic strategy to chemotherapy, which bypasses the need of a functional p53 signalling, based on the direct activation of a p53 miRNA target: miR-34a.

### p53 mutations in MB

Mutated version of p53 with a lower molecular weight, denoted as p47, Δ40p53 or ΔN-p53 have been reported, [37], [38]. This arises due to an alternative translation start site within *TP53*, resulting in a truncated p53 isoform which lacks the N-terminus. The consequence is the expression of a p53 protein without a functional Mdm-2 binding and transactivation domain. However, this variant can still form heterotetramers with full length p53, hence interfering with WT p53 function and act as a downregulator of p53 transactivation [37], [38]. The expression of such truncated p53 can further impact with treatment efficiency. For example, in leukaemia, a low molecular weight form of p53 is inversely correlated with the response to chemotherapy [39]. The presence of an even lower molecular weight p53 forms at 22 kDa has been reported in MB samples, including solid tissue tumours and MB xenografts [35]. We have previously reported that MEB-Med8A cells display a lower p53 band on western-blot [8] and here we show, using a combination of western blot and live cell imaging that the p53 activation is much slower and weaker in the MEB-Med8A cells compared to the p53 WT D283-MED cells (Fig. 2 & 3). In line with the observation mentioned above of truncated p53 acting as an inhibitor of p53 transactivation, we have shown with live cell imaging experiments that re-introduction of p53WT in the MEB-Med8A cells, failed to allow WT p53 stabilisation or Mdm-2 transcription upon etoposide treatment. It is therefore of major importance to be able to therapeutically target MB cells bearing such p53 truncation, by avoiding the need of the p53 signalling.

### miR-34a in tumours and its role in tumour suppression

To circumvent the need of p53, one possibility is the direct activation of one of the p53 target genes involved in either cell cycle arrest or cell death. Yet, whilst p53 activates a complex program involving hundreds of such genes, triggering the expression of only one target is likely to be insufficient to alter cell fate. miRNA expression is regulated by transcription factors in the same way as coding mRNA, and because they can themselves regulate the expression of hundreds of protein targets, they are attractive candidates to inhibit or express. We therefore focused on miR-34a, which is a well described transcriptional target of p53 [22] and, like p53, it also exhibits tumour suppressor activities [40]. Whilst mir-34a mimic triggered ∼30% of cell death in MB and GB cell lines resistant to etoposide, surprisingly no significant cell death was observed for p53WT MB cells. We would have anticipated a stronger effect in p53WT cells due to the activation of the positive feedback on p53 activation [41] and hence increased cell death. Also when the effects of miR-34a mimic expression were measured on miR-34a target protein levels, miR-34a mimic induced similar silencing of SIRT1 and c-Myc in both cell lines (Fig. 5B, D). However, a difference in Bcl-2 silencing was observed, with a more efficient down-regulation in MEB-Med8A cells. This could explain the stronger effect of miR-34a in inducing cell death in MEB-Med8A cells. Altogether our results point to the limited efficacy of miR-34a mimic for brain tumours in clinic in absence of tumour stratification. For non-mutated p53 cells, chemotherapy is likely to be more efficient than direct miRNA activation, yet in absence of a functional p53, miR-34a expression can efficiently mimic, at least in part the effects of etoposide. Moreover, miR-34a mimic might also be used in conjunction with classical chemotherapeutic approaches to increase cell sensitivity in tumours where p53 is functional, as suggested by [30], [42]–[45].

### SIRT1 inhibition

The mechanisms by which miR-34a induce apoptosis in brain tumours happen through down-regulation of multiple oncogenes and pro-survival genes [46]. We here focused on SIRT1, an energy sensor, involved in ageing, metabolism and tolerance to oxidative stress though its deacetylase function on histones and a number of transcription factors, including p53 (for review [47]). Because of its role on down-regulating SIRT1, miR-34a is part of a positive feedback loop acting on p53 [41]. Besides its inhibitory role on p53 transcriptional activity, SIRT1 also contributes to Rb deacetylation, thereby contributing to cell cycle progression [48]. Hence, through both p53 and Rb mechanisms, SIRT1 inhibition results in increase of cell cycle arrest and apoptosis. Indeed, in agreement with other studies, we found that inhibition of SIRT1 by high concentration of nicotinamide [50 mM] reduced cell proliferation even in p53 mutated cells [49]–[51]. However, we found that other SIRT1 inhibitors such as EX527 and sirtinol asserted a small cytotoxic effect on MB cells and that once again a high concentration dose of EX527 [100 µM] was required to cause 50% cell viability after 48 hours treatment in D283-MED cells (Fig. S1). These findings are similar to the one described in [52], where the authors found that EX527 decreased SIRT1 deacetylase activity in epithelial cells but with no effect on cell viability. This result and the need of high concentrations of nicotinamide to induce cell death challenge the idea of using SIRT1 inhibitor alone as a therapeutic agent for brain tumour regression.

In conclusion, miR-34 mimics seem a more appropriate strategy for the treatment of p53 mutated and chemo-resistant brain tumours. The use of miR-34a mimic *in vivo* in several cancer models is encouraging. For example, Kasinski et al have shown that introducing miR-34a in mice with a Kras background prevented tumour formation and progression [53]. Moreover, MRX34 is a synthetic miRNA mimic which has now entered phase 1 clinical trials in patients with primary liver cancer [54]. As mentioned above, the ability of miRNA mimic to target several pathways is a promising way to efficiently impair tumour progression, reducing the potential for tumour adaptation and resistance, as it has been previously observed for targeted therapies. In the case of MB, a possible miR-34a therapy should initially be developed and tested on SHH models of MB, in which p53 mutation remains an important cause of treatment failure and poor outcome.

## Supporting Information

Figure S1 **Vehicle control on cell death and p53-dependent transcription.** (**A**) D283-MED, MHH-Med1 and MEB-Med8A cells were treated with 1/1000 (v/v) DMSO for 6 h and levels of Mdm2 mRNA were assessed by real time qPCR. Results were normalised to cyclophilin A and fold changes relative to the untreated control cells cultured in their complete culture medium. The DMSO used for etoposide dilution has no effect on p53-dependent transcription. (**B**) Cell viability of D283-MED, MHH-Med1 and MEB-Med8A was measured by MTS assay upon treatment at indicated time points with 1/1000 (v/v) DMSO. The values plotted are the optical densities measured from colorimetric MTS reading. There is no influence of the DMSO carrier on cell proliferation and viability.
(TIF)

Figure S2 **Efficiency of cell death induction by SIRT1 inhibitors.** The percentage of cell viability was measured by MTS assay. (**A**) D283-MED cells were treated with EX527 at indicated concentrations for indicated times. (**B**) Same than in (A) for MEB-Med8A cells. (**C**) D283-MED cells treated with Sirtinol [50 or 100 µM] for indicated times. Error bars shown are SD of 6 replicates.
(TIF)
