# Supplementary figures and images for: Mir-34a Mimics Are Potential Therapeutic Agents for p53-Mutated and Chemo-Resistant Brain Tumour Cells
Source: PLoS One. 2014 Sep 24;9(9):e108514. doi: 10.1371/journal.pone.0108514 (PMC4177398; doi:10.1371/journal.pone.0108514)

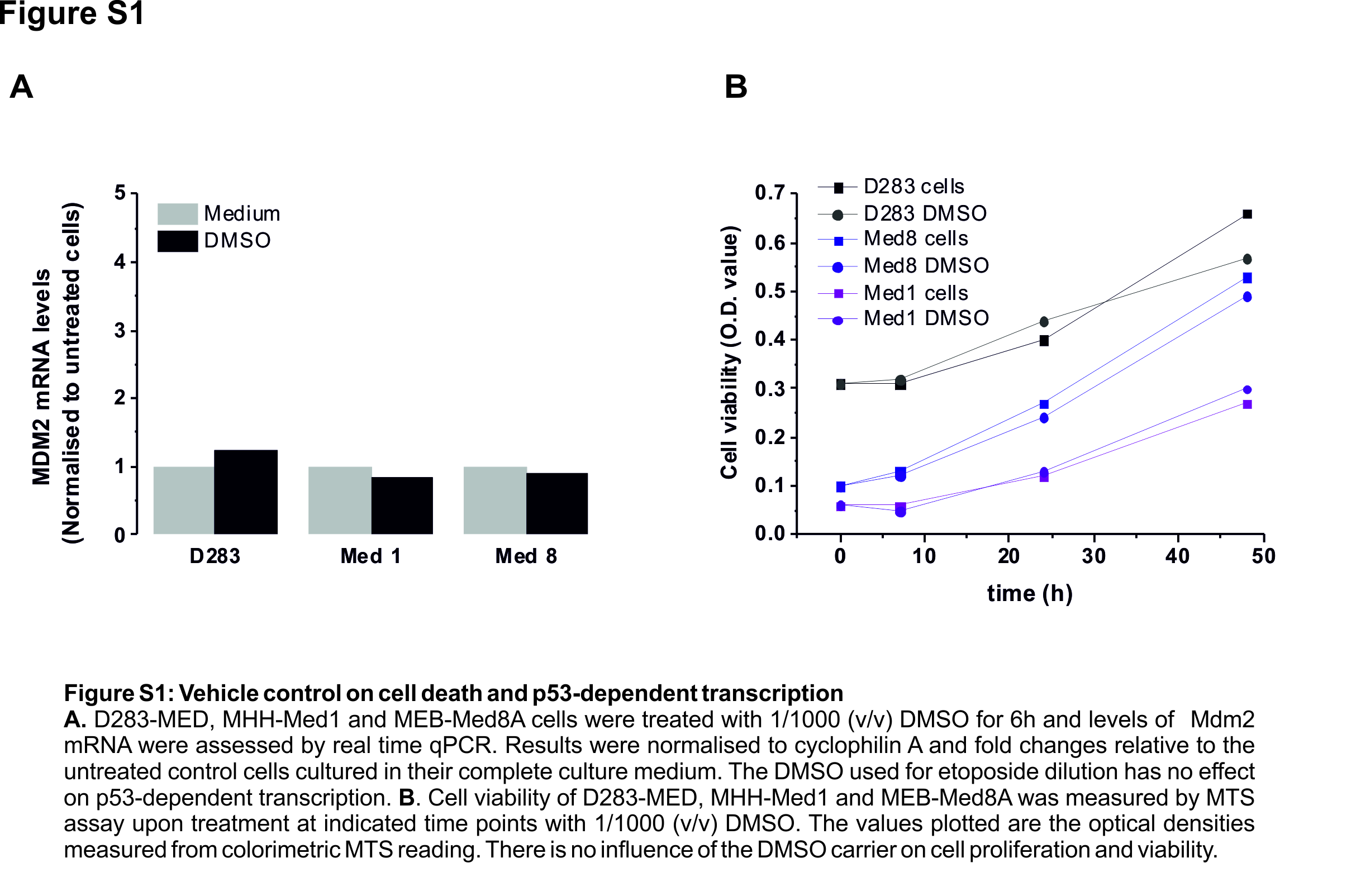

Supplement: Figure S1 — Vehicle control on cell death and p53-dependent transcription. (A) D283-MED, MHH-Med1 and MEB-Med8A cells were treated with 1/1000 (v/v) DMSO for 6 h and levels of Mdm2 mRNA were assessed by real time qPCR. Results were normalised to cyclophilin A and fold changes relative to the untreated control cells cultured in their complete culture medium. The DMSO used for etoposide dilution has no effect on p53-dependent transcription. (B) Cell viability of D283-MED, MHH-Med1 and MEB-Med8A was measured by MTS assay upon treatment at indicated time points with 1/1000 (v/v) DMSO. The values plotted are the optical densities measured from colorimetric MTS reading. There is no influence of the DMSO carrier on cell proliferation and viability. (TIF) [file pone.0108514.s001.tif]

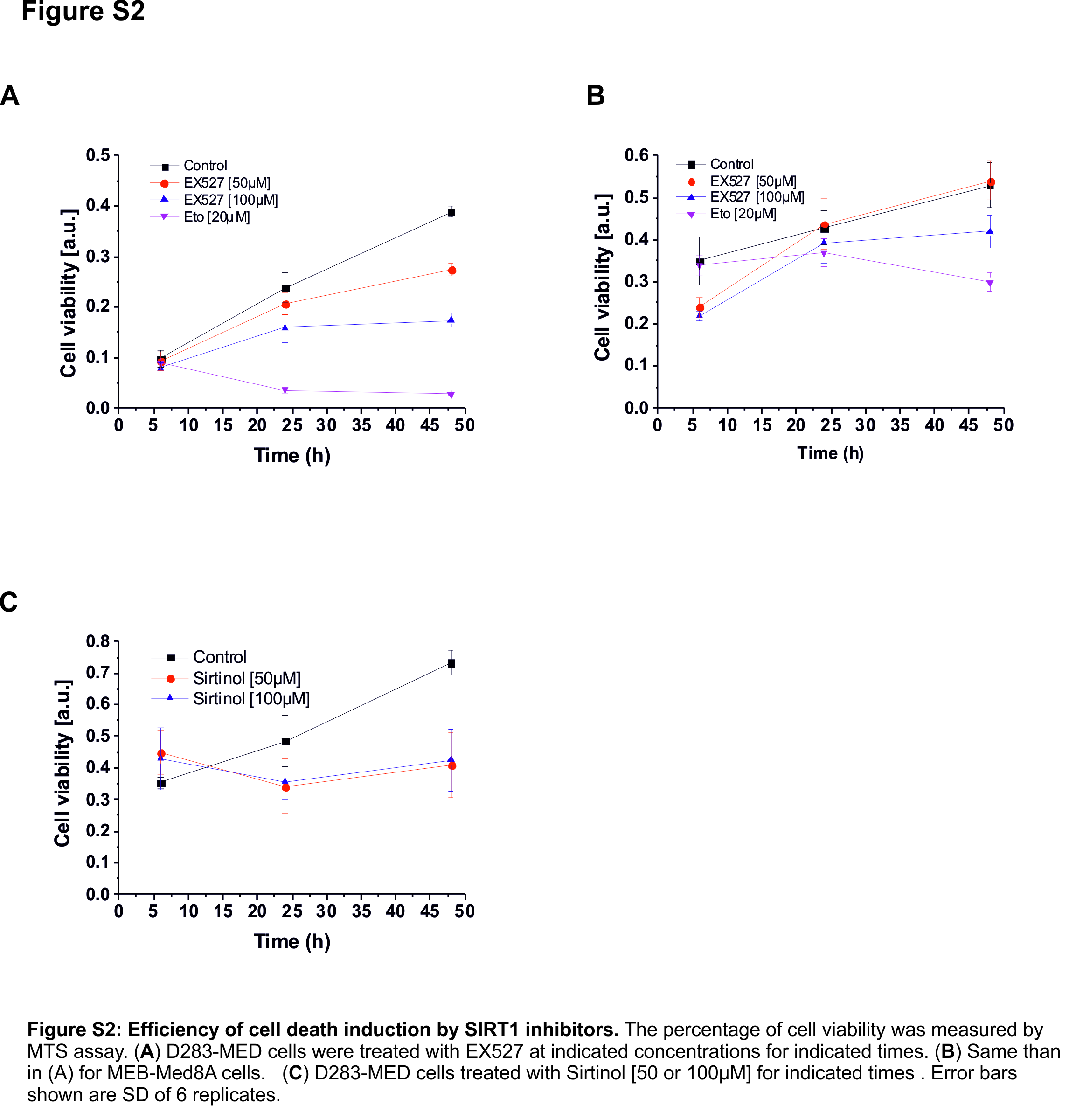

Supplement: Figure S2 — Efficiency of cell death induction by SIRT1 inhibitors. The percentage of cell viability was measured by MTS assay. (A) D283-MED cells were treated with EX527 at indicated concentrations for indicated times. (B) Same than in (A) for MEB-Med8A cells. (C) D283-MED cells treated with Sirtinol [50 or 100 µM] for indicated times. Error bars shown are SD of 6 replicates. (TIF) [file pone.0108514.s002.tif]
